# Elevated Urinary Neutrophil Gelatinase-Associated Lipocalin Is a Biomarker for Lupus Nephritis: A Systematic Review and Meta-Analysis

**DOI:** 10.1155/2020/2768326

**Published:** 2020-06-30

**Authors:** Yueming Gao, Bin Wang, Jingyuan Cao, Songtao Feng, Bicheng Liu

**Affiliations:** Institute of Nephrology, Zhongda Hospital, Southeast University School of Medicine, Nanjing 210009, China

## Abstract

**Objective:**

Lupus nephritis (LN) is a major and severe complication of systemic lupus erythematosus (SLE). Neutrophil gelatinase-associated lipocalin (NGAL), as a promising next-generation biomarker in clinical nephrology, has received extensive attention. However, its diagnostic performance in LN has high variability. Therefore, we performed an updated meta-analysis to further evaluate the diagnostic accuracy of urinary NGAL (uNGAL).

**Materials and Methods:**

PubMed, Embase, and Cochrane Library were searched from inception to October 27, 2019. Meta-analysis was performed with a bivariate random effects model. Additionally, the summary receiver operating characteristic (SROC) curves were established. The sources of heterogeneity were explored by meta-regression, subgroup analysis, and sensitivity analysis. Publication bias was assessed using the Deeks test.

**Results:**

19 articles consisting of 21 eligible studies were included. In diagnosing LN, the estimates (95% confidence interval (CI)) were as follows: sensitivity, 0.84 (0.71-0.91); specificity, 0.91 (0.70-0.98); and the SROC-AUC value, 0.92 (0.90-0.94). In identifying active LN, the estimates were as follows: sensitivity, 0.72 (0.56-0.84); specificity, 0.71 (0.51-0.84); and the AUC value, 0.77 (0.74-0.81). With respect to predicting renal flare, the estimates were as follows: sensitivity, 0.80 (0.57-0.92); specificity, 0.67 (0.58-0.75); and the AUC value, 0.74 (0.70-0.78). For the studies to distinguish proliferative LN, the estimates were as follows: sensitivity, 0.87 (0.66-0.97), and specificity, 0.69 (0.39-0.91). Deeks' funnel plot suggested that there was no significant publication bias.

**Conclusions:**

Our meta-analysis indicates that uNGAL was a useful biomarker for diagnosis, estimation of activity, and prediction of renal flare of LN. In addition, the usefulness of uNGAL to distinguish pathological types of LN needs to be further investigated.

## 1. Introduction

Systemic lupus erythematosus (SLE) is a complex multisystem autoimmune disease characterized by the production of numerous antibodies to cellular components and marked by complicated manifestations, ranging from detectable laboratory abnormalities to multiorgan inflammation and failure [1]. Lupus nephritis (LN), a major risk factor for morbidity and mortality in SLE [2], is a real challenge in the management of SLE due to the lack of effective methods in diagnosing subclinical onset and identifying relapses. Neutrophil gelatinase-associated lipocalin (NGAL, also known as lipocalin-2) is a 25 kDa lipocalin originally purified from human neutrophils [3]. NGAL is an acute-phase glycoprotein secreted in small amounts by neutrophils, epithelial cells, macrophages, hepatocytes, adipocytes, and neurons under physiological conditions, and its expression is significantly increased when it responds to cellular stress [4]. The elevated level of NGAL is associated with injury to epithelial cells in the gastrointestinal tract, respiratory tract, or renal tubules [5]. The relatively small size, secreted pattern, and reliable stability have made it a valuable diagnostic and prognostic biomarker in multiple diseases including acute or chronic kidney diseases [6–8], sepsis [9], cardiovascular diseases [10, 11], inflammatory bowel diseases [4], and cancer [12, 13]. NGAL can be detected in both serum and urine. Urinary biomarkers seem to be more promising than serum biomarkers in the diagnosis of kidney diseases, as the former is derived directly from the inflamed tissue [14].

A previous meta-analysis published in 2015 suggested that uNGAL was a potential biomarker in diagnosing LN and monitoring LN activity [15], but the number of eligible studies was relatively small and did not provide evidence about the role of NGAL in identifying proliferative LN. With accumulating evidence, there is an unmet need for us to perform a systematic review and an updated meta-analysis to further address the usefulness of uNGAL for diagnosis, monitoring, and prediction of LN.

## 2. Materials and Methods

### 2.1. Literature Search

The report of the methods used for this systematic review and meta-analysis was in accordance with the Preferred Reporting Items for Systematic Reviews and Meta-Analyses (PRISMA) consensus statement [16]. Two independent reviewers conducted a comprehensive literature search in the electronic databases including PubMed, Embase, and Cochrane Library up to October 27, 2019. Search strategies included Medical Subject Heading (MeSH) terms and keywords. The MeSH terms were “lupus erythematosus, systemic” and “lupus nephritis”. The keywords included “lupus”, “SLE”, “LN”, “neutrophil gelatinase-associated lipocalin”, “NGAL”, and “lipocalin”. We also searched the combined mode of MeSH or keywords. All retrieval was not restricted by language. In addition, we searched the reference lists of eligible papers manually to identify additional relevant studies. The detailed literature search methods are presented in supplementary Table 1 (Table S1).

### 2.2. Study Selection

The included articles were evaluated by two independent reviewers. Unrelated articles were excluded by reading titles and abstracts of the literatures. If articles were relevant to our research topic, the full texts were carefully read to determine the inclusion or exclusion criteria of the articles. Discrepancies were resolved by discussion or consulting a third investigator. Articles were included if the studies fulfilled the following criteria: (1) the studies were observational studies; (2) the patients were diagnosed according to the American College of Rheumatology (ACR) or Systemic Lupus International Collaborating Clinics (SLICC) classification criteria for SLE; (3) the studies evaluated the diagnostic accuracy of uNGAL concentration in LN vs. non-LN, patients with active LN vs. inactive LN, patients with renal flares vs. without renal flares, and patients with proliferative LN vs. with nonproliferative LN; (4) the studies provided mandatory data from which true-positive (TP), false-positive (FP), false-negative (FN), and true-negative (TN) values could be directly found or calculated; and (5) urine samples were obtained from the spot urine. The exclusion criteria were as follows: (1) studies that were duplicates; (2) studies that were reviews, case reports, meta-analysis, conference abstracts, and animal or cell experiments; (3) studies with irrelevant contents; and (4) studies that did not provide TP, FP, FN, and TN which were used to form a 2 × 2 contingency table.

### 2.3. Data Extraction and Quality Assessment

Two authors independently extracted the data from all the eligible studies, and they were both blind to the relevant contents of the included studies to reduce bias. The following items were extracted from the included studies: (1) basic characteristics of the studies: first author's name, year of publication, study design, region, population type, mean age, percentage of female patients, ethnicity, the method for the NGAL assay, pathological classification criteria, and renal disease activity score, and (2) outcomes of the studies: the optimal cut-off threshold and TP/FP/FN/TN values which were extracted directly or calculated by the Review Manager Software version 5.3 (RevMan 5.3).

The quality assessment of the included studies was performed by the quality assessment tool for diagnostic accuracy studies 2 (QUADAS-2) [17]. The tool is composed of 4 key domains: patient selection, index test, reference standard, and flow and timing. For the evaluation of each domain, the following judgments were used: yes, no, low risk, high risk, and unclear risk. We defined “yes” or “low risk” as 1 score and “no,” “high risk,” or “unclear risk” as 0 score and calculated the total score. RevMan 5.3 was used for the analysis of the risk of bias and applicability concerns.

### 2.4. Statistical Analysis

The diagnostic meta-analysis was performed using Stata version 12.0 software (Stata Corporation, College Station, TX, USA) and Meta-DiSc version 1.4 (XI Cochrane Colloquium, Barcelona, Spain). Heterogeneity was estimated using Cochran's *Q* test and the *I*-squared (*I*^2^) statistical test. *I*^2^ values of 25, 50, and 75% were thought to indicate low, moderate, and high heterogeneity, respectively [18]. If the heterogeneity was significant (*P*_*Q*_ < 0.05 or *I*^2^ > 50%), a random effects model (DerSimonian-Laird method) was used to calculate the pooled sensitivity, specificity, positive likelihood ratio (PLR), negative likelihood ratio (NLR), and diagnostic odds ratio (DOR) with 95% confidence interval (CI); otherwise, a fixed effects model (Mantel-Haenszel method) was used. Moreover, forest plots of sensitivity, specificity, and summary receiver operating characteristic (SROC) with an area under the curve (AUC) value were presented. SROC was used to assess whether there was a “shoulder-arm” pattern or not. A typical “shoulder-arm” pattern would indicate the presence of the threshold effect. Furthermore, the relationship between sensitivity and specificity evaluated by the Spearman correlation coefficient was used to further evaluate the threshold effect. An AUC ≥ 0.70 was defined as a useful risk predictor [19]. Additionally, meta-regression and subgroup analysis were performed to explore the sources of heterogeneity among the included studies. We also performed a sensitivity analysis to examine the stability of our meta-analysis. In addition, the publication bias was assessed by Deeks' funnel plot method, and values of *p* < 0.05 were considered statistically significant.

## 3. Results

### 3.1. Search Results

**Figure 1:**
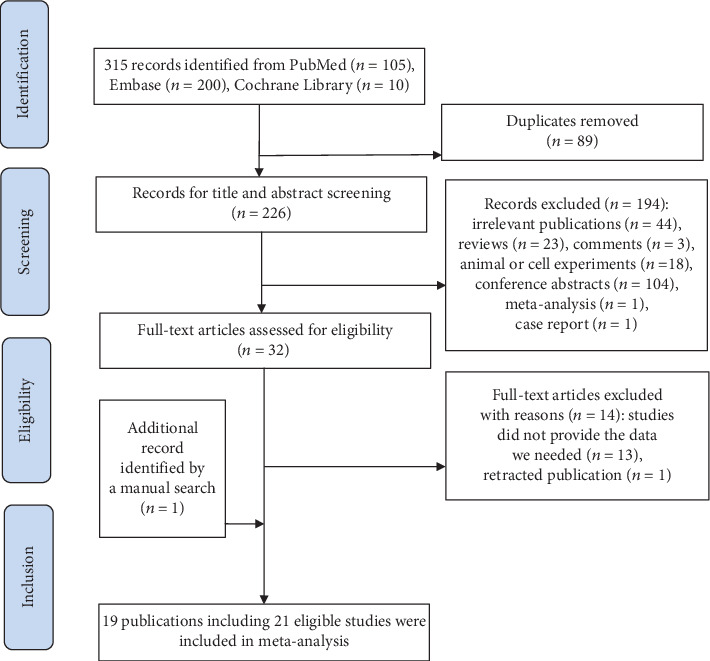
Summary of the literature searching process.

The detailed process of literature searching is illustrated by a flowchart (Figure 1). Initially, 315 articles were identified from electronic databases including PubMed, Embase, and Cochrane Library. By excluding duplicates, 226 articles remained. After a screening of the title and abstract, 194 articles were excluded according to study type and content. The remaining 32 articles were evaluated in detail, of which 13 were removed because they did not provide the data we needed to form a 2 × 2 contingency table. One was removed because it was a retracted publication. In addition, one publication was identified by a manual search. Finally, 19 articles consisting of 21 eligible studies were included in our meta-analysis [20–38].

### 3.2. Characteristics of the Included Studies

The basic characteristics of the 21 eligible studies published in 2006-2019 are listed in Table 1. 11 prospective cohort studies [22–24, 28–31, 35, 36, 38] and 10 cross-sectional studies [20, 21, 25–27, 29, 32–34, 37] were included in our meta-analysis. A total of 1453 patients were enrolled in these studies, including 1269 female patients. Among the 21 eligible studies, 5 were conducted in the United States of America (USA) [20–24], 5 in European countries [23, 29, 30, 35], 5 in Asian countries [25, 27, 28, 32, 38], 5 in African countries [26, 31, 33, 34, 36], and 1 in South American country [37]. Four studies were conducted in children [20, 22, 26, 30], and 17 studies investigated adults [21, 23–25, 27–29, 31–38]. The studies investigated patients from different races, including White, African American, Hispanic, Asian, and others. uNGAL level was detected by the enzyme-linked immunosorbent assay (ELISA) in all studies except the study conducted by Watson et al. [30]. 10 studies [20–23, 25, 27, 33, 37, 38] used the World Health Organization (WHO) classification system for pathological classification of LN, while 8 studies [24, 26, 28, 29, 32, 35, 36] used the International Society of Nephrology/Renal Pathology Society (ISN/RPS) classification system. Three studies [30, 31, 34] did not report the pathological classification criteria.

### 3.3. Quality Assessment

**Figure 2:**
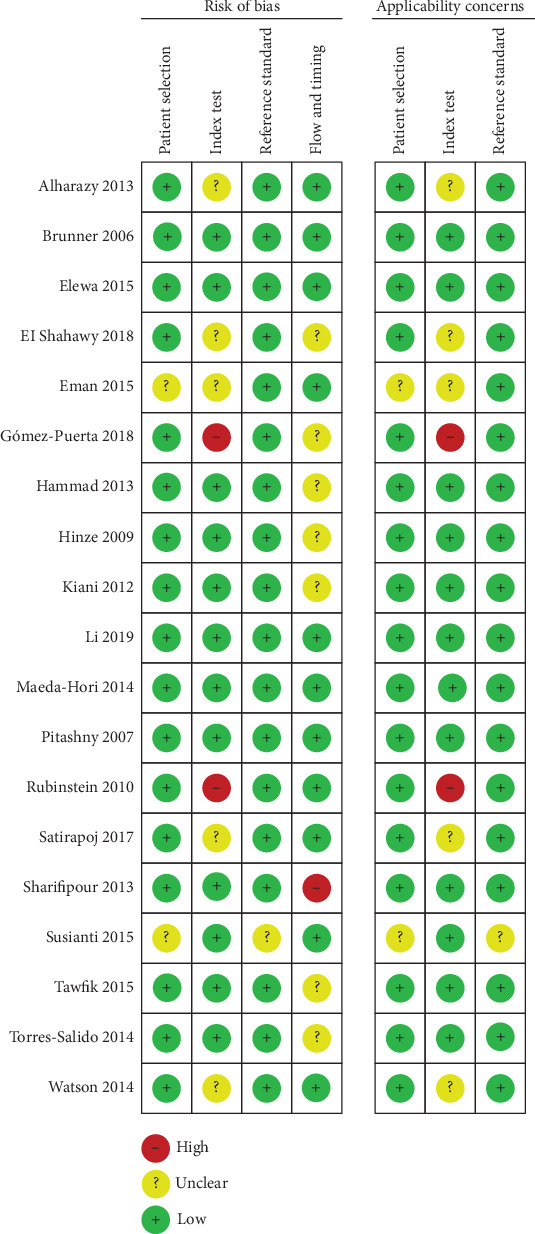
Graph of risk of bias and applicability concerns.

According to the graph of risk of bias and applicability concerns (Figure 2), the included studies had a high risk of bias in terms of the index test, as well as flow and timing, but a low risk of bias in patient selection and reference standard. The unclear risk of bias and the concern regarding the applicability of the patient selection were introduced because 2 studies [32, 34] did not avoid a case-control design. In terms of the index test, only 6 studies [20, 22, 26, 28, 30, 35] were conducted in a blind manner. In addition, 6 studies [23, 25, 30, 35, 37] did not use a prespecified threshold, which may potentially improve the diagnostic performance of uNGAL. In terms of the reference standard, only 7 [20, 22, 26, 28, 30, 34, 35] of the 21 eligible studies explained the results of the reference standard without knowledge of the results derived from the index test. In terms of the flow and timing, 4 studies [33, 35–37] did not use the same gold standard for all subjects, and 6 studies [22, 24, 26, 27, 29] did not include all patients into the diagnostic test. Taken together, the overall quality of these included studies was moderate. The quality assessment scores are listed in Table 1.

### 3.4. Data Synthesis

The data extracted from the 21 eligible studies are presented in Table 2, including TP/FP/FN/TN values, sensitivities, specificities, the optimal cut-off values, and the reference standard for each study. The studies were divided into four parts according to different aims of the relevant studies. Nine studies [20, 21, 27, 32–34, 36–38] reported the ability of uNGAL in the diagnosis of LN; 10 studies [20, 23–25, 28, 29, 31, 35, 37] demonstrated the ability of uNGAL in distinguishing active LN; 6 studies [22, 23, 29–31] showed the diagnostic accuracy of uNGAL in the prediction of renal flare; 2 studies suggested the capacity of uNGAL to identify proliferative LN [26, 33].

#### 3.4.1. Part 1: The Diagnostic Accuracy for uNGAL to Identify LN

**Figure 3:**
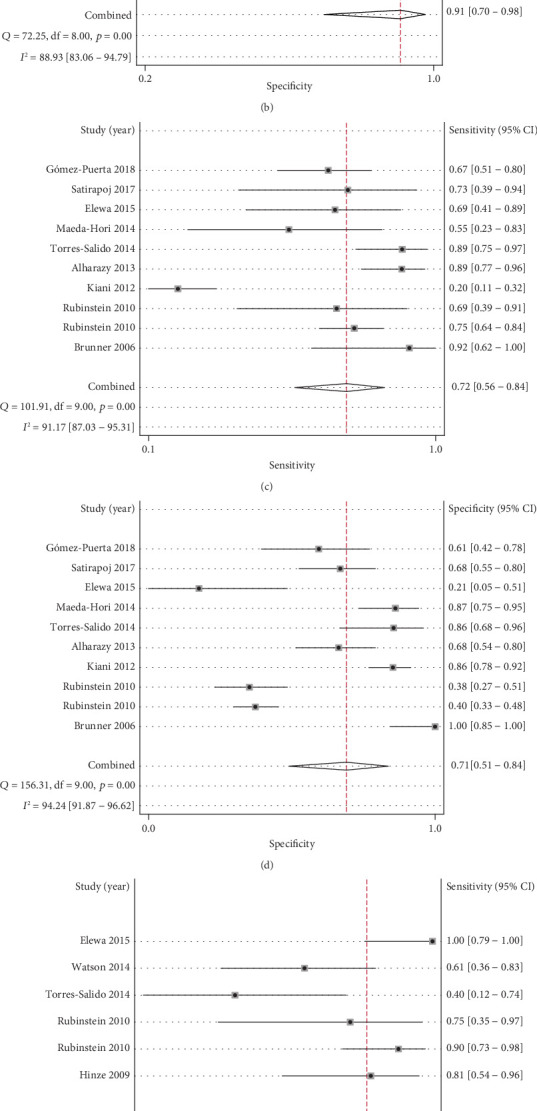
Forest plots for sensitivity and specificity for uNGAL in part 1 to part 3. Forest plot for sensitivity and specificity of uNGAL to identify LN (a, b). Forest plot for sensitivity and specificity of uNGAL to identify active LN (c, d). Forest plot for sensitivity and specificity of uNGAL to predict renal flare (e, f).

As shown in Figure 3 and Table 3, the overall pooled sensitivity of uNGAL for the diagnosis of LN was 0.84 (95% CI, 0.71-0.91) and specificity was 0.91 (95% CI, 0.70-0.98). The overall pooled PLR and NLR were 9.08 (95% CI, 2.31-35.69) and 0.18 (95% CI, 0.09-0.35), respectively, with a DOR of 50.51 (95% CI, 8.15-313.03). Heterogeneity in the pooled sensitivity and specificity was *Q* = 57.96 (*I*^2^ = 86.20%, *p* < 0.001) and *Q* = 72.25 (*I*^2^ = 88.93%, *p* < 0.001), respectively.

**Figure 4:**
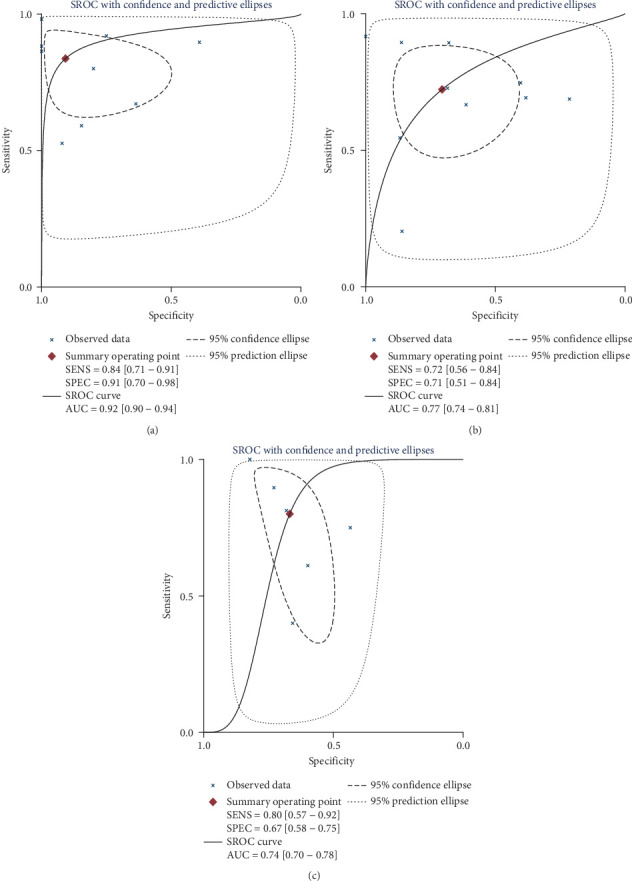
SROC curve for uNGAL in part 1 to part 3. SROC curve of uNGAL to identify LN (a). SROC curve of uNGAL to identify active LN (b). SROC curve of uNGAL to predict renal flare (c).

As shown in Figure 4, the AUC value of the SROC curve was 0.92 (95% CI, 0.90-0.94). The points in the plots did not show a “shoulder-arm” shape, suggesting the absence of the threshold effect. Furthermore, the Spearman correction coefficient between the logit of sensitivity and logit of 1 − specificity of uNGAL was -0.117 (*p* = 0.765), also indicating that there was no threshold effect.

#### 3.4.2. Part 2: The Diagnostic Accuracy for uNGAL to Identify Active LN

As shown in Figure 3 and Table 3, the overall pooled sensitivity and specificity for uNGAL to identify active LN were 0.72 (95% CI, 0.56-0.84) and 0.71 (95% CI, 0.51-0.84), respectively. In addition, the pooled PLR was 2.45 (95% CI, 1.32-4.54), NLR was 0.39 (95% CI, 0.22-0.70), and DOR was 6.24 (95% CI, 2.08-18.68). The heterogeneity was detected in the following indices, including sensitivity (*Q* = 101.91, *I*^2^ = 91.17%, *p* < 0.001) and specificity (*Q* = 156.31, *I*^2^ = 94.24%, *p* < 0.001). As shown in Figure 4, the AUC value of the SROC curve was 0.77 (95% CI, 0.74-0.81), and there was no threshold effect according to the Spearman rank correlation analysis (Spearman correlation coefficient: -0.127, *p* value = 0.726).

#### 3.4.3. Part 3: The Diagnostic Accuracy for uNGAL to Predict Renal Flare

As shown in Figure 3 and Table 3, the overall pooled sensitivity of the 6 eligible studies was 0.80 (95% CI, 0.57-0.92), specificity was 0.67 (95% CI, 0.58-0.75), PLR was 2.41 (95% CI, 1.57-3.72), NLR was 0.30 (95% CI, 0.11-0.79), and DOR was 8.08 (95% CI, 2.02-32.35). The heterogeneity detected in the pooled sensitivity and specificity was *Q* = 18.18 (*I*^2^ = 72.50%, *p* < 0.001) and *Q* = 14.77(*I*^2^ = 66.15%, *p* = 0.01), respectively. As shown in Figure 4, the SROC-AUC value was 0.74 (95% CI, 0.70-0.78) and the graph of the SROC curve was not a “shoulder-arm” shape. Also, there was no threshold effect according to the Spearman rank correlation analysis (Spearman correlation coefficient: -0.771, *p* value = 0.072).

#### 3.4.4. Part 4: The Diagnostic Accuracy for uNGAL to Identify Proliferative LN

As shown in Table 3, the pooled sensitivity for two studies was 0.87 (95% CI, 0.66-0.97), specificity was 0.69 (95% CI, 0.39-0.91), PLR was 2.89 (95% CI, 1.26-6.61), NLR was 0.20 (95% CI, 0.06-0.65), and DOR was 16.42 (95% CI, 2.56-105.37). Of note, the SROC curve could not be constructed for uNGAL to distinguish proliferative LN because of the small number (<4) of relevant studies.

### 3.5. Heterogeneity Analysis

Heterogeneity was significant in all parts of the meta-analysis. Therefore, meta-regression (when the number of the included studies is greater than or equal to 10), subgroup analysis, and sensitivity analysis were conducted to explore possible sources of heterogeneity for part 1 to part 3.

In part 1, as shown in Table 3, four studies [20, 21, 33, 38] formed a subgroup with QUADAS − 2 scores ≥ 13, the pooled sensitivity decreased from 0.84 to 0.81, and specificity increased from 0.91 to 0.95. Specifically, the heterogeneity of sensitivity increased from 86.20% to 89.90%. The heterogeneity of specificity decreased from 88.93% to 62.70%.

In part 2, the following covariates were used as predictor variables in the meta-regression analysis: patient type (children (*n* = 1) or adults (*n* = 9)), design type (prospective cohort study (*n* = 6) or cross-sectional study (*n* = 4)), publication year (2010 and before (*n* = 3) or after 2010 (*n* = 7)), reference standard (renal Systemic Lupus Erythematosus Disease Activity Index (R-SLEDAI) (*n* = 6) or others (*n* = 4)), and quality of study (QUADAS-2 scores < 13 (*n* = 4) or QUADAS-2 scores ≥ 13 (*n* = 6)). The coefficients and *p* value of these variables are listed in supplementary Table 2 (Table S2). Of note, the *p* value of the design type was 0.0284 indicating that it was a potential source of heterogeneity among these studies. Then, subgroup analysis was performed according to the design type, and the results showed that the pooled sensitivity and specificity in the cross-sectional subgroup were higher than those in the prospective cohort subgroup (0.87 vs. 0.57 and 0.82 vs. 0.61, respectively). The heterogeneity of sensitivity and specificity in the cross-sectional subgroup was also lower when compared to the pooled results of the entire ten studies (84.56% vs. 91.17% and 84.04% vs. 94.24%, respectively) (Table 3).

In part 3, based on the reference standard, the pooled sensitivity and specificity in the three studies [22, 23, 31] of the R-SLEDAI subgroup increased from 0.80 to 0.90 and 0.67 to 0.74, respectively, and the heterogeneity in sensitivity and specificity of the R-SLEDAI subgroup decreased from 72.50% to 55.40% and 66.15% to 21.17%, separately (Table 3). As shown in supplementary Figure 1 (Figure S1), after removing the study of Elewa et al. [31], the heterogeneity in sensitivity and specificity was lower than before (65.05% vs. 72.50% and 43.96% vs. 66.15%, respectively).

### 3.6. Publication Bias

**Figure 5:**
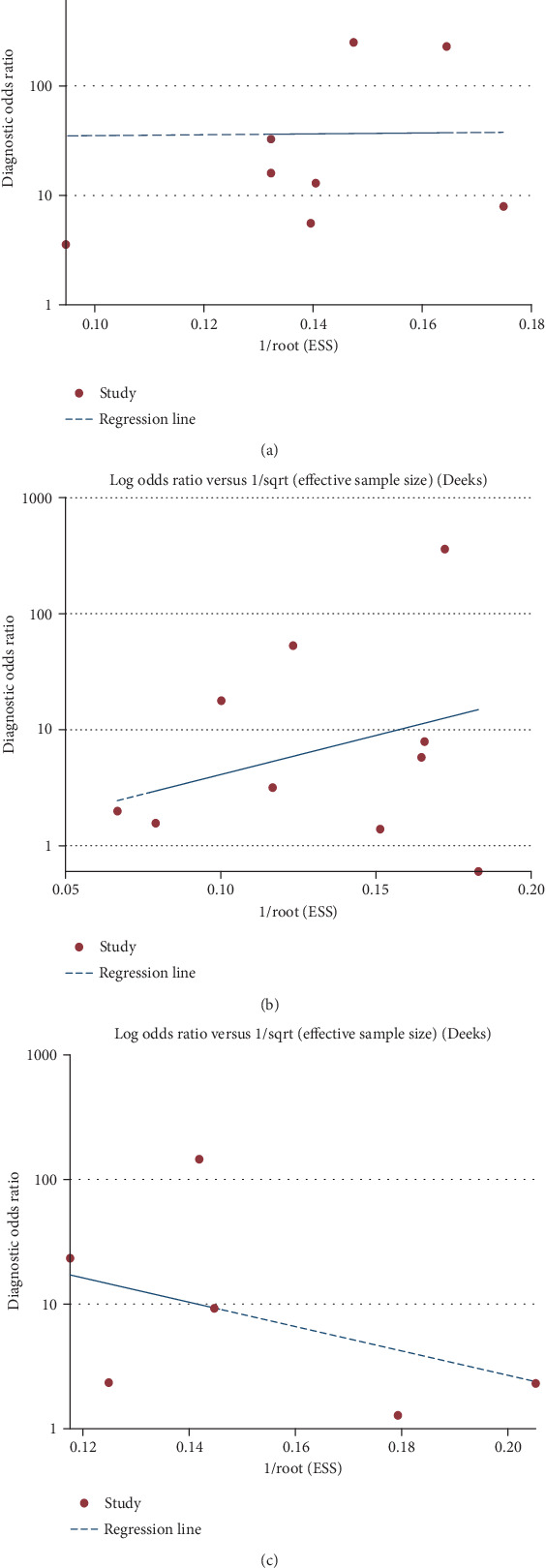
Deeks' funnel plots for part 1 to part 3. Deeks' funnel plots of uNGAL to identify LN (a). Deeks' funnel plots of uNGAL to identify active LN (b). Deeks' funnel plots of uNGAL to predict renal flare (c).

As shown in Figure 5, the evaluation of publication bias according to the Deeks funnel plot asymmetry test showed that there was no potential bias in part 1 to part 3 (*p* value = 0.861, 0.254, and 0.465, respectively).

## 4. Discussion

LN, a severe complication of SLE, poses a real challenge in the management of SLE patients because of the difficulty in early diagnosis and identification of relapses [39]. On the one hand, traditional clinical parameters such as proteinuria, glomerular filtration rate (GFR), urine sediments, anti-dsDNA, and complement levels are not sensitive or specific enough for diagnosis, monitoring of disease activity, and early relapse of nephritis [14]. On the other hand, renal biopsy, as the gold standard for diagnosis and prognosis of LN, is an invasive method and may cause potential complications [40]. Therefore, there is an urgent need for the identification of reliable noninvasive biomarkers with good sensitivity and specificity that contribute to the diagnosis and monitoring of LN. In the past twenty years, NGAL has been the most widely studied biomarker in AKI and has been demonstrated to possess an excellent diagnostic performance. Previous studies have shown that concentrations in urine and serum of NGAL represent sensitive, specific, and highly predictive biomarkers for acute renal injury (AKI) after cardiac surgery [41, 42], in kidney transplantation [43] and critically ill patients [44]. Since 2006, an increasing number of studies have demonstrated the usefulness of urinary NGAL (uNGAL) in the diagnosis and monitoring of LN, but there is a wide range of variability in uNGAL's diagnostic performance. An existing meta-analysis [15] evaluated the diagnostic performance of uNGAL in LN. For the aim of diagnosing LN, it only included 4 eligible studies, and for estimating LN activity, it included 8 studies. But the number of studies has increased since 2015, so we performed an updated meta-analysis to derive a more accurate estimation for the diagnosis and prognosis of LN and also provided evidence for uNGAL to identify proliferative LN.

The main results of our current meta-analysis could be summarized as follows: uNGAL performed well in all parts investigated, with the pooled sensitivity ranging from 0.72 to 0.87 and the specificity ranging from 0.67 to 0.91, respectively. The AUC values of the SROC curves for diagnosing LN, active LN, and renal flare were all beyond 0.70. Of note, meta-analysis of SROC curves revealed a high diagnostic profile for uNGAL to identify LN (AUC value = 0.92). Apart from the valuable diagnostic performance, there was significant heterogeneity in all parts of our meta-analysis. In the meta-analysis of diagnostic tests, the threshold effect is an important source of heterogeneity [45]. In our meta-analysis, we tested the threshold effect in all parts of our meta-analysis and found that there were no obvious threshold effects, which indicated that threshold effects might not be a source of heterogeneity in our meta-analysis. To explore other possible sources of heterogeneity, we conducted meta-regression, subgroup analysis, and sensitivity analysis in part 1 to part 3. After removing the studies with a QUADAS-2 score < 13, the remaining subgroups showed better diagnostic accuracy for the diagnosis of LN, suggesting that the quality of the studies may be a potential source of heterogeneity. In addition, the application of the blinding method, the storage time, and temperature for uNGAL samples might also introduce potential bias as assessed by QUADAS-2. A meta-regression analysis was conducted in part 2 using the following covariates: patient type, design type, publication year, reference standard, and quality of the study, indicating that design type may be a potential source of heterogeneity. The cross-sectional subgroup in distinguishing active LN had better diagnostic accuracy and lower heterogeneity. The R-SLEDAI subgroup in part 3 showed increased sensitivity and specificity, as well as significantly decreased heterogeneity. According to the results of sensitivity analysis, by removing the study of Elewa et al. [31], the heterogeneity decreased and the summary results became more robust. Moreover, Deeks' funnel plots revealed that there was no obvious heterogeneity produced by publication bias.

Apart from uNGAL, serum NGAL was also detected in several included studies [21, 22, 26, 29, 33], most of the studies [21, 26, 33] showed that serum levels of NGAL were not significantly different between LN and controls, one study pointed that serum NGAL levels were statistically different between patients with active LN and those with nonactive SLE, and another study [22] indicated that serum NGAL levels increased significantly before worsening of LN as measured by the BILAG renal score. However, the number of studies exploring the diagnostic accuracy of serum NGAL in LN is relatively small, which still needs to be further evaluated in future studies.

Although uNGAL has been verified to be a satisfactory diagnostic biomarker for LN, identifying new biomarkers or a combination of relevant biomarkers to diagnose and predict LN in a more sensitive and specific way remains an unmet need. Studies have demonstrated pentraxin 3 (PTX3), a regulator of the innate immunity system participating in the tubulointerstitial inflammation, and its level was significantly increased in patients with active LN and might be a biomarker for disease progression [46]. Other biomarkers such as monocyte chemotactic protein 1 [47], ceruloplasmin [48], adiponectin [49], and kidney injury molecule 1 [50] were also verified to be valuable biomarkers in the diagnosis and monitoring of LN. Brunner and his colleagues [51] developed a Renal Activity Index for Lupus (RAIL) based solely on laboratory measures, including uNGAL and other biomarkers, which could accurately reflect histologic LN activity in children. The role of RAIL in the prediction of LN activity in adults was also demonstrated to be excellent, indicating the promising value of the combined biomarkers in the diagnosis and prognosis of LN [52].

Additionally, the diagnostic value of uNGAL still needs to be tested in studies with better quality. Firstly, the studies we included in our meta-analysis are mainly single-center studies; therefore, multicenter studies are urgently needed to confirm the association between uNGAL level and LN and confirm its role in predicting the progression of LN. Secondly, studies including a greater number of patients will gain greater insight into the potential usefulness of urinary lipocalin-2 in patients with LN. Additionally, studies should predetermine their cut-offs according to the values proposed in the present review, in order to improve the quality and reliability of these studies. Furthermore, it is also recommended that the combination of laboratory biomarkers-RAIL needs to be assessed in more validation cohorts.

## 5. Limitations

This study was limited by certain factors. Firstly, the number of the eligible studies for identifying proliferative LN was too small to establish a SROC curve, so we presented the diagnostic accuracy merely by description. Secondly, there was significant heterogeneity in all parts of the meta-analysis, and meta-regression, subgroup analysis, and sensitivity analysis could only explain part of the sources of heterogeneity. Thirdly, many of the studies included did not use a blinding method, and the reference standards might vary from different studies, which might introduce potential bias in the summary of results. Lastly, the fact that uNGAL/Cr instead of absolute values of uNGAL was measured in some of the included studies might also play a role in the presence of heterogeneity.

## 6. Conclusion

In conclusion, our updated meta-analysis indicates that uNGAL was a useful biomarker for diagnosis, estimation of activity, and prediction of renal flare of LN and its diagnostic value for diagnosing LN was superior to those under other settings. In addition, the usefulness of uNGAL to distinguish pathological types of LN needs to be further investigated.

## Figures and Tables

**Table 1:** Basic characteristics of the included studies in the meta-analysis.

First author's name, year [reference]	Design	Number of patients	Region	Population type	Mean age (years)	Women (%)	Ethnicity (%)	NGAL assay	Pathological classification criteria	QUADAS-2 score
Brunner, 2006 [20]	CS	35	USA	Children	11.6 (4)^a^	32 (91)	—	ELISA (Denmark)^d^	WHO	16
Pitashny, 2007 [21]	CS	70	USA	Adults	35 (28-44)^a^	58 (83)	African American (41), Hispanic (56), others (3)	ELISA (Denmark)^d^	WHO	15
Hinze, 2009 [22]	PC	111	USA	Children	15.9 ± 3.4^b^	89 (90)	White (49.5), African American (32.4), Asian (12.6), Hispanic (11.7)	ELISA (Denmark)^d^	WHO	14
Rubinstein, 2010 [23]	PC	107	USA (Bronx)	Adults	41 (12)^a^	97 (91)	Hispanic (47), Black (46), White (2), others (4)	ELISA (Denmark)^d^	WHO	12
Rubinstein, 2010 [23]	PC	35	UK (London)	Adults	41 (13)^a^	34 (97)	White (49), Black (23), Asian (14), Southeast Asian (9), others (6)	ELISA (Denmark)^d^	WHO	12
Kiani, 2012 [24]	PC	107	USA	Adults	41^c^	97 (91)	Black (51), White (36), Asian (4), Hispanic (4), others (5)	ELISA (R&D)^e^	ISN/RPS	13
Alharazy, 2013 [25]	CS	100	Malaysia	Adults	36.90 ± 10.62^b^	92 (92)	Malay (41), Chinese (55), Indian (4)	ELISA (R&D)^e^	WHO	12
Hammad, 2013 [26]	CS	33	Egypt	Children	10 (8-12)^a^	25 (76)	—	ELISA (R&D)^e^	ISN/RPS	15
Sharifipour, 2013 [27]	CS	52	Iran	Adults	26.38 ± 5.86^b^	44 (85)	—	ELISA (R&D)^e^	WHO	12
Torres-Salido, 2014 [29]	CS	123	Spain	Adults	33 (27-42)^a^	109 (89)	White (93), Hispanic (2), African American (2), others (2)	ELISA (Denmark)^d^	ISN/RPS	13
Torres-Salido, 2014 [29]	PC	45	Spain	Adults	31 (23-41)^a^	39 (87)	White (89), Hispanic (7), African American (2), others (2)	ELISA (Denmark)^d^	ISN/RPS	13
Watson, 2014 [30]	PC	64	UK	Children	11.3 (8.0-13.3)^a^	51 (80)	Caucasian (50), Black African (14), Indian (9), Caribbean (8), Asian (6), Bangladeshi (4), mixed race (3), Chinese (2), others (2)	Architect assay^f^	—	14
Maeda-Hori, 2014 [28]	PC	64	Japan	Adults	44.1 ± 17.0^b^	46 (72)	Asian (100)	ELISA (R&D)^e^	ISN/RPS	16
Elewa, 2015 [31]	PC	30	Egypt	Adults	32.84 ± 10.47^b^	29 (97)	—	ELISA (Boster)^g^	—	15
Susianti, 2015 [32]	CS	50	Indonesia	Adults	28.88 ± 8.58^b^	48 (96)	Javanese (92), Madurese (8)	ELISA (RayBiotech)^h^	ISN/RPS	10
Tawfik, 2015 [33]	CS	35	Egypt	Adults	30 (24-37)^a^	31 (89)	—	ELISA (R&D)^e^	WHO	13
Youssef, 2015 [34]	CS	44	Egypt	Adults	30.12 ± 6.8^b^	35 (80)	—	ELISA^i^	—	11
Satirapoj, 2017 [35]	PC	68	UK	Adults	31.7 ± 10.0^b^	66 (97)	—	ELISA (R&D)^e^	ISN/RPS	14
El Shahawy, 2018 [36]	PC	70	Egypt	Adults	26.94 ± 6.01^b^	67 (96)	—	ELISA (R&D)^e^	ISN/RPS	10
Gómez-Puerta, 2018 [37]	CS	120	Colombia	Adults	32.8 ± 12.1^b^	105 (88)	Mestizo (76.7), Afro Latin-American (21.6), Caucasian (1), Colombian Amerindian (1)	ELISA (R&D)^e^	WHO	10
Li, 2019 [38]	PC	90	China Taiwan	Adults	37.1 ± 1.3^b^	75 (83)	—	ELISA (R&D)^j^	WHO	15

NGAL: neutrophil gelatinase-associated lipocalin; CS: cross-sectional study; PC: prospective cohort study; USA: the United States of America; UK: the United Kingdom; ELISA: enzyme-linked immunosorbent assay; WHO: World Health Organization classification system; ISN/RPS: the International Society of Nephrology/Renal Pathology Society classification system. QUADAS-2: quality assessment tool for diagnostic accuracy studies 2; “yes” or “low concern”: 1 score; “no” or “high concern” or “unclear”: 0 score. ^a^Median (interquartile range (IQR)). ^b^Mean ± SD. ^c^Median. (Denmark)^d^: Bioporto, Gentofte, Denmark. (R&D)^e^: R&D Systems, Minneapolis, MN, USA. Architect assay^f^: Abbott Laboratories, Abbott Park, IL, USA. (Boster)^g^: Boster Biological Technology Co., Ltd. (RayBiotech)^h^: ELHLipocalin2-001, RayBiotech® Inc., Norcross, GA, USA. ELISA^i^: BioVendor, Asheville, NC28806, USA; (R&D)^j^: Quantikine: R&D Systems, Inc.

**Table 2:** Summary of data available for meta-analysis.

Study	Cut-off point	Reference standard	TP	FP	FN	TN	Sensitivity (95% CI)	Specificity (95% CI)
Part 1: the diagnostic accuracy for uNGAL to identify LN in SLE								
Brunner et al. 2006 [20]	0.6 ng/mg Cr	Biopsy	15	0	2	18	0.88 (0.64, 0.99)	1.00 (0.81, 1.00)
Pitashny et al. 2007 [21]	28 ng/mg Cr	Biopsy	10	3	9	35	0.53 (0.29, 0.76)	0.92 (0.79, 0.98)
Sharifipour et al. 2013 [27]	0.39 ng/mg Cr	Biopsy	26	14	3	9	0.90 (0.73, 0.98)	0.39 (0.20, 0.61)
Susianti et al. 2015 [32]	446.3 pg/ml	Biopsy	40	4	10	16	0.80 (0.66, 0.90)	0.80 (0.56, 0.94)
Tawfik et al. 2015 [33]	20 ng/mg Cr	Biopsy	13	2	9	11	0.59 (0.36, 0.79)	0.85 (0.55, 0.98)
Eman et al. 2015 [34]	13.2 ng/dl	Biopsy	19	0	3	22	0.86 (0.65, 0.97)	1.00 (0.85, 1.00)
El Shahawy et al. 2018 [36]	13.66 ng/ml	Biopsy	46	5	4	15	0.92 (0.81, 0.98)	0.75 (0.51, 0.91)
Gómez-Puerta et al. 2018 [37]	11.98 ng/ml	Biopsy	51	16	25	28	0.67 (0.55, 0.77)	0.64 (0.48, 0.78)
Li et al. 2019 [38]	80 ng/ml	Biopsy	53	0	1	36	0.98 (0.90, 1.00)	1.00 (0.90, 1.00)
Part 2: the diagnostic accuracy for uNGAL to identify active LN								
Brunner et al. 2006 [20]	0.6 ng/mg Cr	R-SLEDAI	11	0	1	23	0.92 (0.62, 1.00)	1.00 (0.85, 1.00)
Rubinstein et al. 2010 [23]	11.7 ng/ml	R-SLEDAI	62	104	21	70	0.75 (0.64, 0.84)	0.40 (0.33, 0.48)
Rubinstein et al. 2010 [23]	11.7 ng/ml	BILAG2004	9	42	4	26	0.69 (0.39, 0.91)	0.38 (0.27, 0.51)
Kiani et al. 2012 [24]	0.3 ng/mg Cr	SLICC	13	15	51	92	0.20 (0.11, 0.32)	0.86 (0.78, 0.92)
Alharazy et al. 2013 [25]	91.25 ng/mg Cr	R-SLEDAI	42	17	5	36	0.89 (0.77, 0.97)	0.68 (0.54, 0.80)
Torres-Salido et al. 2014 [29]		R-SLEDAI	34	4	4	25	0.90 (0.75, 0.97)	0.86 (0.68, 0.96)
Maeda-Hori et al. 2014 [28]		BAI	6	7	5	46	0.55 (0.23, 0.83)	0.87 (0.75, 0.95)
Elewa et al. 2015 [31]		R-SLEDAI	11	11	5	3	0.69 (0.41, 0.89)	0.21 (0.05, 0.51)
Satirapoj et al. 2017 [35]	28.08 ng/ml	UPCR	8	18	3	39	0.73 (0.39, 0.94)	0.68 (0.55, 0.80)
Gómez-Puerta et al. 2018 [37]		R-SLEDAI	30	12	15	19	0.67 (0.51, 0.80)	0.61 (0.42, 0.78)
Part 3: the diagnostic accuracy for uNGAL to predict renal flare								
Hinze et al. 2009 [22]		R-SLEDAI	13	15	3	32	0.81 (0.54, 0.96)	0.68 (0.53, 0.81)
Rubinstein et al. 2010 [23]	13.6 ng/ml	R-SLEDAI	26	13	3	35	0.90 (0.73, 0.98)	0.73 (0.58, 0.85)
Rubinstein et al. 2010 [23]	13.6 ng/ml	BILAG2004	6	13	2	10	0.75 (0.35, 0.97)	0.44 (0.23, 0.66)
Torres-Salido et al. 2014 [29]	0.421 ng/ml Cr	Proteinuria	4	12	6	23	0.40 (0.12, 0.74)	0.66 (0.48, 0.81)
Watson et al. 2014 [30]	30 ng/ml	pBILAG	11	59	7	88	0.61 (0.36, 0.83)	0.60 (0.51, 0.68)
Elewa et al. 2015 [31]		R-SLEDAI	16	8	0	37	1.00 (0.79, 1.00)	0.82 (0.68, 0.92)
Part 4: the diagnostic accuracy for uNGAL to identify proliferative LN								
Hammad et al. 2013 [26]	10.07 ng/mg Cr	Biopsy	11	3	1	7	0.92 (0.62, 1.00)	0.70 (0.35, 0.93)
Tawfik et al. 2015 [33]	18 ng/ml	Biopsy	9	1	2	2	0.82 (0.48, 0.98)	0.67 (0.09, 0.99)

TP: true positive; FP: false positive; FN: false negative; TN: true negative; CI: confidence interval; uNGAL: urinary neutrophil gelatinase-associated lipocalin; LN: lupus nephritis; R-SLEDAI: renal Systemic Lupus Erythematosus Disease Activity Index; SLICC: Systemic Lupus International Collaborating Clinics renal activity score; BAI: biopsy activity index; UPCR: urinary protein/creatinine ratio; pBILAG: the global paediatric version of the British Isles Lupus Assessment Group 2004 index; BILAG2004: British Isles Lupus Assessment Group 2004 index.

**Table 3:** Pooled accuracy indices, subgroup analysis, and sensitivity analysis of uNGAL.

Number of studies	Pooled sensitivity (95% CI)	*I* ^2^ (%)	Pooled specificity (95% CI)	*I* ^2^ (%)	Pooled PLR (95% CI)	Pooled NLR (95% CI)	Pooled DOR (95% CI)	AUC
Part 1: the diagnostic accuracy for uNGAL to identify LN								
All (9)	0.84 (0.71, 0.91)	86.20	0.91 (0.70, 0.98)	88.93	9.08 (2.31, 35.69)	0.18 (0.09, 0.35)	50.51 (8.15, 313.03)	0.92
*QUADAS* − 2 ≥ 13 subgroup (4)	081 (0.81, 0.91)	89.90	0.95 (0.89, 0.98)	62.70	10.75 (2.65, 43.57)	0.21 (0.07, 0.67)	60.96 (6.09, 610.22)	1.00
Part 2: the diagnostic accuracy for uNGAL to identify active LN								
All (10)	0.72 (0.56, 0.84)	91.17	0.71 (0.51, 0.84)	94.24	2.45 (1.32, 4.54)	0.39 (0.22, 0.70)	6.24 (2.08, 18.68)	0.77
Cross-sectional subgroup (4)	0.87 (0.71, 0.95)	84.56	0.82 (0.57, 0.94)	84.04	4.89 (1.63, 14.67)	0.16 (0.06, 0.43)	30.60 (4.30, 217.68)	0.92
Prospective cohort subgroup (6)	0.57 (0.37, 0.75)	89.83	0.61 (0.40, 0.79)	94.92	1.46 (1.12, 1.91)	0.71 (0.56, 0.88)	2.07 (1.39, 3.08)	0.62
Part 3: the diagnostic accuracy for uNGAL to predict renal flare								
All (6)	0.80 (0.57, 0.92)	72.50	0.67 (0.58, 0.75)	66.15	2.41 (1.57, 3.72)	0.30 (0.11, 0.79)	8.08 (2.02, 32.35)	0.74
R-SLEDAI subgroup (3)	0.90 (0.80, 0.96)	55.40	0.74 (0.66, 0.81)	21.70	3.41 (2.31, 5.03)	0.17 (0.07, 0.40)	20.75 (6.13, 70.29)	
Five studies without Elewa 2015	0.73 (0.54, 0.86)	65.05	0.63 (0.56, 0.70)	43.96	1.97 (1.41, 2.77)	0.43 (0.23, 0.82)	4.55 (1.74, 11.87)	0.68
Part 4: the diagnostic accuracy for uNGAL to identify proliferative LN								
All (2)	0.87 (0.66, 0.97)		0.69 (0.39, 0.91)		2.89 (1.26, 6.61)	0.20 (0.06, 0.65)	16.42 (2.56, 105.37)	

CI: confidence interval; PLR: positive likelihood ratio; NLR: negative likelihood ratio; DOR: diagnostic odds ratio; AUC: area under the curve. CS: cross-sectional study; PC: prospective cohort study. QUADAS-2: quality assessment tool for diagnostic accuracy studies 2; R-SLEDAI: renal Systemic Lupus Erythematosus Disease Activity Index.

## Supplementary Materials

Supplementary Materials Figure S1: plot of sensitivity analysis in part 3. Sensitivity analysis plot of uNGAL to predict renal flare. The results showed that the study of Elewa in part 3 might influence the robustness of the meta-analysis. Table S1: the detailed literature search methods for the meta-analysis. Table S2: meta-regression for part 2.
