# Supplementary material for: Elevated Urinary Neutrophil Gelatinase-Associated Lipocalin Is a Biomarker for Lupus Nephritis: A Systematic Review and Meta-Analysis
Source: Biomed Res Int. 2020 Jun 30;2020:2768326. doi: 10.1155/2020/2768326 (PMC7346103; doi:10.1155/2020/2768326)
Supplement: Supplementary Materials — Figure S1: plot of sensitivity analysis in part 3. Sensitivity analysis plot of uNGAL to predict renal flare. The results showed that the study of Elewa in part 3 might influence the robustness of the meta-analysis. Table S1: the detailed literature search methods for the meta-analysis. Table S2: meta-regression for part 2. [file 2768326.f1.docx]

**Supplementary materials**

**Figure S1.** Sensitivity analysis for part 3.


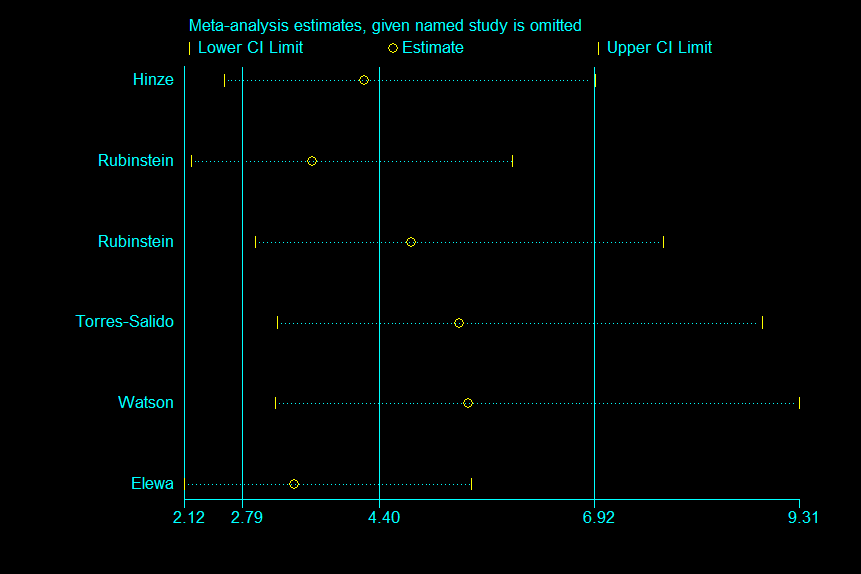


Supplementary Materials Figure 1. Plot of sensitivity analysis in part 3. Sensitivity analysis plot of uNGAL to predict renal flare. The results showed that the study of Elewa in part 3 might influence the robustness of the meta-analysis.

| **Table S1. The detailed literature search methods for the meta-analysis.** | | |
| --- | --- | --- |
| **Search methods in PubMed** | | |
| **Search** | **Query** | **Items found** |
| #5 | Search(((((("Lupus Erythematosus, Systemic" [Mesh]) OR "Lupus Nephritis"[Mesh]) OR lupus) OR SLE) OR LN)) AND ((((neutrophil gelatinase-associated lipocalin) OR NGAL) OR lipocalin) | 105 |
| #4 | Search(((neutrophil gelatinase-associated lipocalin) OR NGAL) OR lipocalin | 10278 |
| #3 | Search (((("Lupus Erythematosus, Systemic"[Mesh]) OR "Lupus Nephritis"[Mesh]) OR lupus) OR SLE) OR LN | 110447 |
| #2 | Search "Lupus Nephritis"[Mesh] | 6202 |
| #1 | Search "Lupus Erythematosus, Systemic"[Mesh] | 57998 |
| **Search methods in EMBASE** | | |
| **Search** | **Query** | **Items found** |
| #6 | #4 AND #5 | 200 |
| #5 | 'neutrophil gelatinase-associated lipocalin':ab,ti OR 'ngal':ab,  ti OR 'lipocalin':ab,ti | 9176 |
| #4 | #1 OR #2 OR #3 | 161215 |
| #3 | 'sle':ab,ti OR 'ln':ab,ti OR 'lupus':ab,ti | 139299 |
| #2 | 'lupus erythematosus nephritis'/exp | 15372 |
| #1 | 'systemic lupus erythematosus'/exp | \| 93683 \| \| --- \| |
| **Search methods in Cochrane Library** | | |
| **Search** | **Query** | **Items found** |
| #6 | #4 And #5 | 10 |
| #5 | (neutrophil gelatinase-associated lipocalin): ti,ab,kw OR (NGAL): ti,ab,kw OR (lipocalin) : ti,ab,kw | 807 |
| #4 | #1 OR #2 OR #3 | 4516 |
| #3 | (lupus):ti,ab,kw OR (SLE):ti,ab,kw OR (LN):ti,ab,kw | 4516 |
| #2 | MeSH descriptor: [Lupus Nephritis] explode all trees | 218 |
| #1 | MeSH descriptor: [Lupus Erythematosus, Systemic] explode all trees | 898 |
| **Additional research identified by manual search** | | 1 |

Supplementary Materials Table 1. The detailed literature search methods for the meta-analysis. PubMed, EMBASE and Cochrane Library were searched from inception to October 27, 2019. Search strategies included Medical Subject Heading (MeSH) terms and keywords. The MeSH terms were “lupus erythematosus, systemic” and “lupus nephritis”. The keywords included “lupus”, “SLE”, “LN”, “neutrophil gelatinase-associated lipocalin”, “NGAL” and “lipocalin”. In addition, one article was identified by searching the reference lists of eligible papers manually. Finally, the literature search identified a total of 316 articles.

| **Table S2. Meta-Regression for part2.** | | | | | |
| --- | --- | --- | --- | --- | --- |
| **Meta-Regression (Inverse Variance weights) (1)** | | | | | |
| Var | Coeff | Std.Err. | p-value | RDOR | (95%CI) |
| Cte | 5.076 | 3.1228 | 0.2025 | — | — |
| S | 0.092 | 0.3934 | 0.8293 | — | — |
| patient | -0.353 | 3.8428 | 0.9326 | 0.70 | (0.00, 143853.10) |
| design | -3.741 | 1.7519 | 0.1224 | 0.02 | (0.00, 6.26) |
| Year | -1.757 | 2.0014 | 0.4446 | 0.17 | (0.00, 100.69) |
| reference | -1.045 | 1.4326 | 0.5186 | 0.35 | (0.00, 33.59) |
| quality | 2.023 | 1.6026 | 0.2960 | 7.56 | (0.05, 1240.82) |
| **Meta-Regression (Inverse Variance weights) (2)** | | | | | |
| Var | Coeff | Std.Err. | p-value | RDOR | (95%CI) |
| Cte | 4.804 | 1.6670 | 0.0449 | — | — |
| S | 0.089 | 0.3223 | 0.7962 | — | — |
| design | -3.803 | 1.2198 | 0.0356 | 0.02 | (0.00, 0.66) |
| Year | -1.912 | 1.0920 | 0.1548 | 0.15 | (0.01, 3.06) |
| reference | -0.992 | 1.2325 | 0.4660 | 0.37 | (0.01, 11.36) |
| quality | 2.128 | 1.0636 | 0.1160 | 8.40 | (0.44, 160.97) |
| **Meta-Regression (Inverse Variance weights) (3)** | | | | | |
| Var | Coeff | Std.Err. | p-value | RDOR | (95%CI) |
| Cte | 3.756 | 1.0114 | 0.0138 | — | — |
| S | -0.053 | 0.2484 | 0.8392 | — | — |
| design | -3.136 | 0.8603 | 0.0148 | 0.04 | (0.00, 0.40) |
| Year | -1.737 | 1.0275 | 0.1516 | 0.18 | (0.01, 2.47) |
| quality | 1.997 | 0.9999 | 0.1023 | 7.37 | (0.56, 96.29) |
| **Meta-Regression (Inverse Variance weights) (4)** | | | | | |
| Var | Coeff | Std.Err. | p-value | RDOR | (95%CI) |
| Cte | 2.529 | 0.7458 | 0.0147 | — | — |
| S | -0.042 | 0.2752 | 0.8829 | — | — |
| design | -2.425 | 0.8088 | 0.0241 | 0.09 | (0.01, 0.64) |
| quality | 1.089 | 0.9336 | 0.2879 | 2.97 | (0.30, 29.16) |
| **Meta-Regression (Inverse Variance weights) (5)** | | | | | |
| Var | Coeff | Std.Err. | p-value | RDOR | (95%CI) |
| Cte | 3.009 | 0.6486 | 0.0024 | — | — |
| S | -0.225 | 0.2363 | 0.3724 | — | — |
| design | -2.231 | 0.8106 | 0.0284 | 0.11 | (0.02, 0.73) |
| Var: variables; Coeff: coeﬃcient; Std.Err.: standard error; RDOR: relative diagnostic odds ratios;  CI: confidence interval. | | | | | |

Supplementary Materials Table 2. Meta-regression for part 2. Patient type, design type, publication year, reference standard, and quality of study were used as predictor variables in the meta-regression analysis. The coeﬃcients and p-value of these variables were listed in the table. The p value of design type was 0.0284 indicating that it was a potential source of heterogeneity among these studies.
